# Maternal diet during pregnancy and adaptive changes in the maternal and fetal pancreas have implications for future metabolic health

**DOI:** 10.3389/fendo.2024.1456629

**Published:** 2024-09-23

**Authors:** David J. Hill, Thomas G. Hill

**Affiliations:** ^1^ Lawson Health Research Institute, St. Joseph’s Health Care, London, ON, Canada; ^2^ Departments of Medicine, Physiology and Pharmacology, Western University, London, ON, Canada; ^3^ Oxford Centre for Diabetes, Endocrinology, and Metabolism, Wellcome Centre for Human Genetics, University of Oxford, Oxford, United Kingdom

**Keywords:** nutrition, pregnancy, fetus, pancreas, insulin, programming

## Abstract

Fetal and neonatal development is a critical period for the establishment of the future metabolic health and disease risk of an individual. Both maternal undernutrition and overnutrition can result in abnormal fetal organ development resulting in inappropriate birth size, child and adult obesity, and increased risk of Type 2 diabetes and cardiovascular diseases. Inappropriate adaptive changes to the maternal pancreas, placental function, and the development of the fetal pancreas in response to nutritional stress during pregnancy are major contributors to a risk trajectory in the offspring. This interconnected maternal-placental-fetal metabolic axis is driven by endocrine signals in response to the availability of nutritional metabolites and can result in cellular stress and premature aging in fetal tissues and the inappropriate expression of key genes involved in metabolic control as a result of long-lasting epigenetic changes. Such changes result is insufficient pancreatic beta-cell mass and function, reduced insulin sensitivity in target tissues such as liver and white adipose and altered development of hypothalamic satiety centres and in basal glucocorticoid levels. Whilst interventions in the obese mother such as dieting and increased exercise, or treatment with insulin or metformin in mothers who develop gestational diabetes, can improve metabolic control and reduce the risk of a large-for-gestational age infant, their effectiveness in changing the adverse metabolic trajectory in the child is as yet unclear.

## 1 Introduction

**Figure 1:**
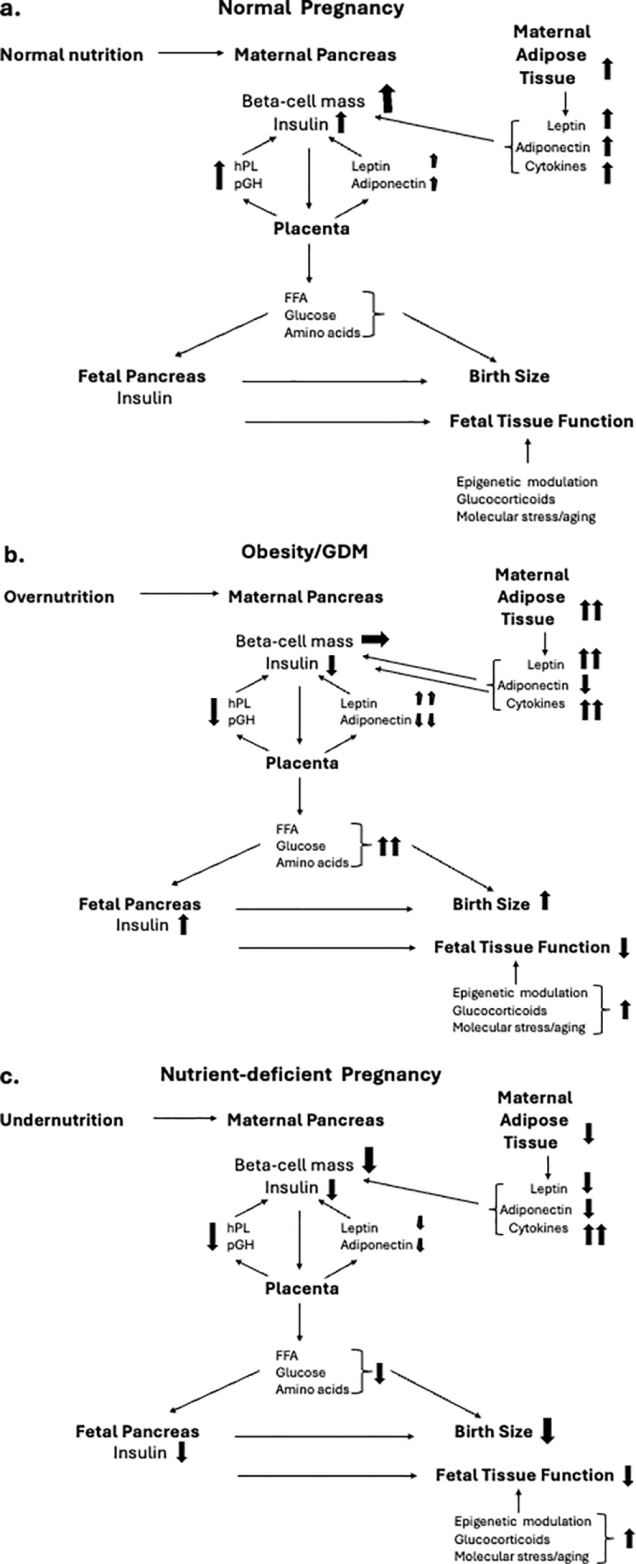
Inter-relationships between maternal nutrition during pregnancy and hormonal interactions between the maternal endocrine pancreas, placenta and fetal pancreas, and their implications for birth size and organ function. Directional changes are shown in schematics for, a) normal nutritional intake, b) obese pregnancy, and c) nutrient-deficient pregnancy. **(A)** During normal pregnancy there is an increase in insulin resistance in response the placentally-derived hormone, placental growth hormone (pGH). The insulin resistance is amplified by increased secretion of leptin and resistin from both the maternal adipose tissue and the placenta, together with adipose-derived cytokines that contribute to maternal tissue metabolic and immune stress. The cellular stress of the maternal endocrine pancreas and other tissues is maintained at non-pathological levels by a parallel increase in adiponectin secretion from both the maternal adipose tissue and the placenta. The diabetogenic pressure of the insulin resistance of pregnancy is countered by an increased secretion of human placental lactogen (hPL) from the placenta which promotes an increase in pancreatic beta cell mass (BCM) and glucose-stimulated insulin secretion (GSIS). The net impact of these changes is to maintain a high and constant level of glucose flux and transport of free fatty acids (FFA) and amino acids across the placenta from mother to fetus. Optimal nutrient delivery enables the fetal pancreas to maintain a regulated release of insulin which acts as a major tissue growth factor to ensure birth size within the normal range. Fetal organ maturation occurs at appropriate developmental stages under the intrinsic regulation of epigentic control of gene expression and tissue aging with modulation by fetal hormones such as glucocorticoids. **(B)** Maternal pre-gestational obesity together with excessive pregnancy weight gain, often developing into GDM, results in elevated cellular stress as a result of increased leptin and cytokine release with a lowering of adiponectin secretion. Synthesis and release of pGH is relatively reduced as is hPL, limiting the adaptive increase in maternal BCM and insulin release. This results in maternal hyperglycemia and increased nutrient transport to the fetus. Fetal hyperglycemia stimulates hyperinsulinemia resulting in fetal overgrowth while fetal hyperlipidemia fuels pathologic adipose deposition, together resulting in large for gestational age birthweight. Elevated fetal glucocorticoid levels and cytokines result in altered epigenetic control of fetal tissue maturation contributing to birth complications such as respiratory distress. Dysfunctional epigenetic control of key genes associated with metabolic control contribute to childhood obesity and adult metabolic disease. **(C)** Undernutrition during pregnancy can result in insufficient gain in maternal adipose tissue, reduced leptin and adiponectin release, elevated cytokine-driven cellular stress and impaired placental function. The mobilization of maternal glucose for transfer to the fetus is impaired as is the adaptive increase to BCM during pregnancy. A reduced efficiency and temporal stability of nutrient transfer across the placenta limits fetal insulin release and optimal fetal tissue growth resulting in small-for-gestational age birthweight. Elevated cellular stress and glucocorticoid levels result in aberrant epigenetic control of key genes associated with metabolic control and endocrine pancreatic function, contributing to adult metabolic disease.

Embryonic and fetal development is a critical window in establishing the future phenotype and functional efficiency of organs and tissues following birth (1). Often referred to as fetal programming, or the developmental origins of adult diseases, maternal nutrition is a major determinant of newborn and adult health (2). The focus in developed countries concerns the impact of maternal obesity on both maternal and child health (3). However, maternal undernutrition remains an issue in war zones and as a consequence of climate change in certain areas of the world. In this narrative review we focus on interactions between the maternal pancreas, placenta, and fetal pancreas during pregnancy and how these are compromised by maternal under- or overnutrition (**Figure 1**).

Pre-gestational maternal obesity and excessive maternal weight gain are both risk factors for a large-for-gestational age (LGA) infant, but also for miscarriage or still-birth (4, 5). The Hyperglycemia and Adverse Pregnancy Outcomes (HAPO) study demonstrated that blood glucose subsequent to exaggerated insulin resistance as experienced by mothers with higher body mass index (BMI) during pregnancy positively correlated with newborn weight and cord blood C-peptide levels (6, 7). The development of gestational diabetes mellitus (GDM) amplifies the risk of an LGA infant further (8), and both maternal obesity and GDM contribute to excess newborn adiposity (9) and a higher risk of childhood obesity (10–13) and subsequent type 2 diabetes (T2D) and cardiovascular disease (14, 15). This axis of altered fetal development also involves changes to placental growth and function with altered expression of genes regulating trophoblast cell proliferation (16). The placenta secretes both leptin and adiponectin into the maternal circulation, the former being increased in obese pregnancies and contributing to impaired maternal glucose-stimulated insulin release (GSIS), adding to the risk of GDM and fetal hyperglycemia (17, 18). Free fatty acid transfer across the placenta to the fetus is elevated in obese pregnancy resulting in increased adipose deposition in the offspring at birth as a result of fetal hyperinsulinemia (19).

The impact of maternal undernutrition on birth size and future health has been extensively documented with low birth weight being associated with the risk of adult T2D, hypertension and coronary heart disease (20–22). The Dutch famine in 1944-1945 lasting 6 months under wartime conditions was shown to alter the risk trajectory of chronic diseases in the offspring of women pregnant during that period. Early gestational exposure to malnutrition was associated with a greater risk of offspring schizophrenia, depression and coronary heart disease, while any period of exposure increased the risks of future T2D (23). Understanding the mechanisms linking fetal undernutrition to metabolic trajectory following birth have benefited from animal models such as reduced calorie maternal diet, specific reduction of dietary protein, or the restriction of utero-placental blood flow (24). In most cases the resulting metabolic phenotype appears only in later life (25).

## 2 The maternal pancreas during pregnancy

Pregnancy represents an increasingly high metabolic demand on the mother. There is an increase in blood volume, heart rate and cardiac output, in addition to systemic increases in tissue oxidative and inflammatory stress and a faster shortening of DNA telomeres, a measure of tissue aging (26). Metabolic stress is also exacerbated by increasing insulin resistance in maternal tissues in second and third trimester (27). A 50-60% decrease in insulin sensitivity occurs with increasing gestation and insulin-mediated whole-body disposal of glucose decreases by 50% due to a decrease in post-insulin receptor signaling and reduced cellular cycling of the GLUT4 glucose transporter (reviewed in 28). A measure of the strength of the insulin resistance associated with pregnancy can be understood from nutritional and physical activity behavioural intervention studies attempting to prevent the onset of GDM. Behavioural modification that resulted in a greater than 3 kg reduction in gestational weight gain caused only a 5% decrease in insulin resistance (29). Placentally-derived growth hormone is primarily responsible for decreasing insulin sensitivity in maternal tissues such as adipose, liver and muscle through signaling via the growth hormone receptor, resulting in altered insulin receptor second messenger signaling to create relative insulin resistance when compared to the pre-pregnancy state (30). Maternal insulin resistance is also driven by increased levels of leptin during pregnancy (31). As a counter-regulation to the insulin resistance of pregnancy the syncytiotrophoblast increases the synthesis and release of placental lactogen (PL) into the maternal circulation throughout gestation, a primary action of which is to facilitate an increase in maternal beta-cell mass (BCM) to increase the capacity for GSIS (32). Placental lactogen and placental growth hormone work in concert to regulate the maternal metabolism for the benefit of both mother and fetus (33).

Pancreatic beta-cells have a high capacity for proliferation during fetal development and early childhood, but proliferative potential declines rapidly following puberty to a basal replacement rate of only-1-2% in adulthood in rodent species, and lower still in human (34–36). However, the pancreatic response to cellular stress during pregnancy is to re-activate beta-cell proliferation, increasing both BCM and increase the capacity for insulin synthesis. This pregnancy-associated increase in BCM has been documented during human pregnancy (37, 38). The beta-cell fractional area within the pancreas in pregnant women in second and third trimester was shown to more than double compared to pancreata from non-pregnant women, being associated with larger islets rather than an increased number (37). A second study using a tissue cohort gathered from pregnant women mainly in first trimester found a smaller, 1.4-fold increase in beta-cell fractional area with an increased number of small islets during pregnancy, and evidence of neogenesis of new beta-cells from progenitors adjacent to the pancreatic ducts (38). The differences between these reports may reflect the ontogeny of beta-cell adaptation of pregnancy with the initial response being the generation of new, small islets by neogenesis which subsequently become larger islets with a mature endocrine cell architecture. Lactogenic hormone treatment of isolated human islets from non-pregnant donors, using either hPL or prolactin, of isolated human islets from non-pregnant donors was shown to increase beta-cell proliferation and GSIS (39). Other placentally-derived peptides may also exert trophic effects on the maternal beta-cells, such as kisspeptin (40), leptin (41) and resistin. In addition to placenta, the adipokine resistin is synthesized and released by the maternal liver, adipose and pancreatic islets with circulating levels peaking in third trimester (42), and being abnormally elevated in obese mothers and those with GDM (43). Resistin has been found to induce the expression of Suppressor of cytokine signaling 3 (SOCS3) resulting in a decrease in insulin release, as well as insulin sensitivity in target metabolic tissues (44, 45).

Similarly, in rodents the BCM in increased by two-to-three-fold over the course of pregnancy (46, 47) and has allowed mechanistic pathways to be identified in a manner not possible in human pregnancy. The presence of PL in the maternal circulation can directly facilitate the re-entry of the normally quiescent beta-cells into the proliferative cycle, while the insulin resistant state of pregnancy also contributes to beta-cell hypertrophy through increased demand for insulin release (48, 49). A heterogeneity of beta-cell phenotypes exist in the pancreas and proliferation during pregnancy may originate from a number of sub-populations. For instance, the Flattop (FLTP) gene modulates islet endocrine cell polarity, and FLTP-positive beta-cells increase as a percentage of BCM in mice with advancing age until adulthood, where they represent 80% of beta-cells (50). FLTP-negative beta-cells have a greater proliferative potential and could, perhaps, selectively increase in number during the adaptive changes during pregnancy. The increase in BCM may also involve the functional maturation of resident beta-cell progenitor cell populations (51). Cell lineage tracing experiments during mouse pregnancy showed that up to a quarter of new beta-cells could derive from resident progenitor cells within the islets of Langerhans (52, 53). Several reports have identified the presence of islet progenitor beta-cells that express some insulin but are poorly responsive to glucose since expression of the glucose-transporter 2 (Glut2) is low (54). These Ins^+^Glut2^LO^ cells exist in both the mouse and human pancreas, and can differentiate both *in vitro* and *in vivo* to form functional beta-cells in response to hyperglycemic stress (55, 56). The Ins^+^Glut2^LO^ cell abundance is significantly increased within mouse islets in mid-gestation, which directly precedes the increase in overall beta-cell proliferation at GD12 (57). Ins^+^Glut2^LO^ cells have a higher rate of proliferation *in situ* when compared to mature beta-cells, associated with a higher expression of the transcription factor pancreatic and duodenal homeobox (*Pdx*)1 (58). More generally, beta-cell expansion in the maternal mouse pancreas is associated with a specific transcription factor expression signature with increased expression of Hepatocyte nuclear factor-4 (*Hnf-4*)α, Forkhead box protein M1 (*Foxm1*) and MAF bZIP transcription factor B (*MafB*), all being associated with functional cell maturity. Interestingly, deletion of *Hnf-4α* from beta-cells prevented the pregnancy-associated increase in BCM and resulted in glucose intolerance (59), whilst experimental inactivation of Foxm1 blocked the ability of PL or prolactin to initiate beta-cell proliferation (60). Similarly, deletion of the *MafB* gene, which is more highly expressed in progenitor/immature beta-cells, resulted in a decrease in proliferative capacity during pregnancy (61).

In mice, two molecular forms of PL exist, PL-1 being synthesized early in gestation with a peak in the maternal circulation on gestational day (GD) 10, and PL-2 which increases subsequently and remains high until parturition (62). Placental lactogen promotes beta-cell replication through binding to, and activation of, the prolactin receptor (PRLR) (63). Prolactin receptor activation allows engagement with the Janus kinase (JAK)2 signaling pathway followed by phosphorylation and translocation of Signal transducer and activator of transcription (STAT)5 to the beta-cell nucleus and a range of gene transcriptional changes (64). Pregnant mice carrying a null mutation of the PRLR showed glucose intolerance and a failure to undergo adaptive changes in BCM (63). Conversely, over-expression of PL resulted in an increased BCM with associated hypoglycemia (65). The actions of PL on beta-cell proliferation can also be indirect by promoting a paracrine release of serotonin from the beta-cells during pregnancy, in addition to increased expression of the serotonin receptor (66, 67). Serotonin enhances proliferation of beta-cells by binding to and activation of the 5-HT2B receptor. Serotonin secretion by beta-cells is higher in pregnant rat islets than in non-pregnant controls, and its inhibition restricts BCM expansion during pregnancy, resulting in glucose intolerance (68).

Maternal pre-gestational obesity or excess gestational weight gain can impair adaptive changes in the maternal endocrine pancreas in response to pregnancy. Pre-existing obesity is associated with relative insulin resistance and this can amplify the insulin resistance subsequently associated with pregnancy, particularly in the metabolically-active tissues such as adipose, liver and skeletal muscle (69). This can be modeled in rodents through dietary manipulation, such as feeding a high fat diet (HFD) with or without a high sugar content (HFSD) (70). The pregnant dam fed HFSD showed elevated fasting blood glucose from mid-pregnancy until term compared to non-pregnant controls, accompanied by tissue insulin resistance. HFSD diet during pregnancy also resulted in a maternal phenotype of hypercholesterolemia and elevated circulating inflammatory cytokines (71). HFD combined with a low dose of streptozotocin (STZ) to partially destroy the beta-cell population (72) resulted in a greater gestational weight gain and elevated blood glucose, insulin, and leptin levels, and a decrease in circulating adiponectin; mimicking many of the features of pre-gestational obesity in human pregnancy (73, 74).

Obesity in pregnancy can lead to macrophage infiltration and the release of cytotoxic cytokines such as Interleukin (IL)-1β from white adipose tissue (75) associated with increased leptin production and leptin resistance at the hypothalamus. The latter can result in increased appetite which further compromises maternal weight gain (76). Conversely, adiponectin secretion from adipose tissue in the obese mother is decreased (77) resulting in a net increase in lipolysis resulting in high circulating fatty acid levels and a lipotoxicity of the maternal pancreatic beta-cells. When coupled with persistent hyperglycemia this environment can result in gluco-lipotoxicity of the beta-cells resulting in oxidative and endoplasmic-reticular stress with a resulting impairment of GSIS, further amplifying maternal and fetal hyperglycemia with resulting fetal overgrowth (78–81). Hyperleptinemia and leptin resistance have been shown to decrease beta-cell proliferation and GSIS during pregnancy in rodent models of maternal overnutrition (81, 82). A reduction in circulating adiponectin in obese pregnancy is also associated with the development of GDM and may impact on the adaptive increase in BCM and function, since adiponectin has been shown to increase beta-cell proliferation in mouse islets (83). Complete genetic deletion of adiponectin resulted in a failure to adequately increase BCM during mouse pregnancy, causing glucose intolerance (84). Placental expression of leptin is increased in women with GDM as is placental responsiveness to leptin, resulting an increased nutrient transfer to the fetus and an increased risk of fetal overgrowth (76, 85). A high pre-gestational BMI and/or excessive gestational weight gain are associated with elevated circulating maternal levels of proinflammatory cytokines, including Tumour necrosis factor (TNF)-α, IL-1β, and IL-6 (86, 87). Increased inflammatory cytokine presence further contributes to beta-cell dysfunction, since exposure of isolated human or mouse islets to IL-1β resulted in impaired GSIS associated with beta-cell de-differentiation and loss of function (82). Thus, maternal obesity can create a multi-faceted shift in nutritional metabolic balance, adipokine and cytokine presence which impairs the adaptive response of the maternal pancreas to increase BCM and insulin release, increasing the risk of maternal hyperglycemia, GDM, and fetal overgrowth.

Chronic maternal undernutrition during pregnancy in rodents through feeding of a low protein (LP) diet resulted in a decreased maternal islet cell area but without a change in pancreatic insulin content or glucose tolerance (88, 89). However, we and others found that exposure of mice to low protein diet throughout pregnancy until term resulted in a relative reduction in maternal weight, glucose intolerance, and impaired GSIS associated with a deficiency in intracellular signaling pathways mediating insulin synthesis and release, such as protein kinase-C (90, 91). In a similar model, greater insulin release was observed at lower glucose concentrations than would normally occur during pregnancy following exposure to LP diet with no change in release at higher concentrations, compared to control rats fed a normal diet (92). This overall reduction in glucose sensitivity of the maternal beta-cells during undernutrition has been linked with mitochondrial dysfunction and reduced Ca^2+^ efflux from beta-cells (93, 94). Certain micronutrients in the LP diet may be particularly responsible for the impaired maternal GSIS since a reduction in tryptophan content alone impaired the increase in BCM in pregnant mice with a reduction in glucose tolerance (68). Tryptophan is essential for the synthesis of serotonin, which is itself a beta-cell mitogen. The effects of undernutrition on maternal BCM and function may be mediated, in part, through the placenta since the junctional zone of the mouse placenta, which contains the PL-expressing endocrine trophoblast cells and other prolactin-like molecules, was smaller following exposure to LP diet, which might result in a lesser PL-dependent increase in maternal BCM (95).

## 3 Maternal nutrition and fetal pancreas function

Maternal obesity, with or without GDM, is associated with hyperinsulinemia and excess adiposity in the offspring (96, 97). It is unclear if the predominant driver of this is maternal hyperglycemia, and consequently fetal hyperglycemia during gestation, excess lipid transfer to the fetus, or both. In rodent models of maternal overnutrition such as feeding a HFD there are abnormalities in endocrine pancreatic morphology in the offspring with a decrease in BCM at birth compared to control-fed dams (98), although this is not consistently associated with hyperinsulinemia (98, 99). Whether an extended period of HFD was utilized pe-pregnancy, or HFD was applied during gestation only, appears to influence fetal insulin secretion at term. Prolonged HFD in mice resulted in elevated circulating insulin levels in the newborn, an elevated BCM and a greater GSIS in the neonates when compared with control-fed dams (100). When HFD was administered during gestation alone circulating insulin levels were not increased in the newborn. Prolonged maternal feeding of HFD in mice can alter the balance of islet endocrine cell development at birth with a greater representation of glucagon-secreting alpha-cells, contributing to impaired glucose tolerance as adults (101). To better separate the importance of maternal glucose versus hyperlipidemia in the mother on the metabolic profile of the offspring, STZ was administered pre-gestationally without the complication of maternal obesity. This resulted in offspring with increased BCM and elevated circulating insulin (102, 103). When adult, these animals became glucose intolerant with loss of beta-cell function suggestive of beta-cell exhaustion (104). Fetal hyperglycemia in the absence of maternal hyperglycemia during pregnancy was achieved in rats by infusing glucose into the vasculature of one uterine horn for the last 48 hours of gestation, while using the opposing horn as control (105). The hyperglycemic fetuses had higher serum insulin levels and an increased BCM, and when these islets were isolated from the fetal pancreata they showed reduced viability *in vitro*. Transcriptomic analysis of the fetal islets revealed a downregulation of genes associated with BCM expansion such as Regenerating gene (*Reg*)3b and *Reg3g* and fibroblast growth factor receptor 3 (*Fgfr3*). The hyperglycemic fetuses subsequently exhibited hyperglycemia and glucose intolerance as adults.

Changes in the placental transfer of nutrients during maternal obesity and/or diabetes may underlie the resulting pancreatic phenotype of the newborn. Expression of the major glucose transporter in the placenta, GLUT1, was reported to be increased in mothers with pre-gestational diabetes compared to non-diabetic mothers, with placental expression and peptide levels being positively correlated with birth weight (106). Similarly, placental expression of key regulators of placental fatty acid transfer to the fetal circulation were altered in mothers with pre-gestational diabetes (107). These included significant increases in the expression of fatty acid translocase (CD36), fatty acid binding proteins, and fatty acid transport protein 4 (FATP4). Fetal lipid presence has been correlated with beta-cell development, since in humans long chain n-3 polyunsaturated fatty acid (PUFA) levels were positively associated with cord plasma proinsulin levels, while n-6 PUFAs were negatively correlated (108). PUFAs have been shown to regulate islet size in the fetal mouse (109). Prolonged pre-gestational obesity was generated in transgenic mice with a placenta-specific gene knockout of adipose triglyceride lipase (*Atgl*) (100). This transgenic model limited fatty acid availability to the fetuses and reversed the increase in BCM and circulating insulin levels seen in the term fetus and neonate of control mice, suggesting that excess free fatty acid presence in the fetal circulation is a contributor to higher insulin release. In an attempt to represent pre-gestational obesity in a non-human primate model a high calorie diet was supplied to Japanese macaques for a minimum of two years prior to pregnancy, compared to a control diet (110). As early as post-weaning the offspring of animals given the Western-style diet showed hypersecretion of insulin from islets in response to a glucose challenge, *in vivo* and *in vitro*, compared to controls, with increased expression of Pdx1 and genes associated with endoplasmic reticular and oxidative stress. In summary, prolonged pre-gestational maternal obesity results in increased transfer of both glucose and lipids to the fetus and results in an a greater BCM and insulin release, possibly related to a temporal advancement of beta-cell maturation. This is likely to compromise beta-cell resilience to a metabolic challenge in later life, resulting in an increased risk of T2D.

While several documented clinical studies of nutritional deprivation during pregnancy exist, such as for the Dutch Hunger Winter (111), longitudinal analyses of the effects of nutrient deprivation on fetal pancreatic development have required animal models of undernutrition, such as the feeding of a LP diet throughout pregnancy, or extended to weaning (112–114). LP diet has been applied continuously until parturition, or given in tertile periods of gestation only (115). Moderate nutritional insults such as LP diet have remarkably little impact on maternal weight gain during pregnancy in rodents with a reduction only apparent in the final 2-3 days of gestation, whilst litter size is normal or near normal (116).

Exposure to a LP diet during gestation resulted in a small lowering of mean birth size in mice or rats but body weights were no different from control diet-fed animals by the time of weaning (117). However, there was a significant reduction in pancreatic weight at birth (118). Within the neonatal pancreas both BCM and mean islet size were significantly lower in offspring from LP-fed dams compared with control diet-fed mothers, and when islets of Langerhans were isolated in late gestation from fetuses exposed to LP diet they exhibited a lower basal and GSIS compared to control islets. The rate of beta-cell proliferation was also lower and the incidence of beta-cell apoptosis higher in islets from LP-exposed offspring at birth (119), together with a reduced microvascular density within the islets of Langerhans (120). The deficit in BCM at birth could be related to a number of developmental changes including local peptide growth factor production, the pro-inflammatory cytokine environment, or beta-cell oxidative stress (121). The changes to islet function could also represent disrupted developmental timing in functional differentiation *in utero*, as the transcription factor expression profile in the pancreas from LP-exposed mice was more representative of mature beta-cell function at birth, whilst control animals showed a more immature profile (122). Also, the ability of beta-cells from newborns exposed to LP diet *in utero* to form pseudo-islets and re-express insulin was impaired, as was the expression of transcription factors associated with beta-cell differentiation such *Pdx1*, Paired box (*Pax*)6 and Neurogenin (*Ngn*)3 (123). The amino acid taurine has been shown to be essential for normal development of the pancreatic beta-cells in the fetus, and taurine levels within the fetal pancreas are significantly depleted following maternal LP diet (124). Supplementation of a LP diet with taurine alone could reverse the detrimental effects on BCM and GSIS in offspring in rodent models (125). The taurine receptor, TauT, is expressed by beta-cells and taurine and beta-alanine both enhance GSIS through an increase in intracellular Ca^2+^ and cAMP (126). The actions of taurine on GSIS were additive to those of the incretin, glucagon-like peptide-1 (GLP-1). These findings suggest that the reduction in essential amino acids within LP diet may represent a determining factor in the compromised endocrine pancreatic function in the fetus.

## 4 Adaptations of the endocrine pancreas to the extra-uterine environment

The functional maturation of the beta-cells in rodent species occurs predominantly between birth and weaning. During gestation the fetus experiences little fluctuation in circulating glucose concentrations due to maternal and placental buffering. Consequently, beta-cells at birth are highly sensitive to amino acid stimulation of glucose secretion but are poorly glucose responsive (127). Neonatal beta-cells exhibit a relatively high basal insulin secretion at low glucose concentrations compared to adult islet, but a limited additional insulin release at high concentration of glucose (128). Analysis of dynamic GSIS on the day of birth in mice showed that islets exhibited both first and second phase insulin release at low glucose concentrations (2.8mM), while at postnatal day 15 islets released no insulin in response to low glucose (129). This suggests a reduced glucose threshold for GSIS at birth compared to more mature beta-cells. Further analysis showed that this change in glucose threshold had occurred by postnatal day 9. Studies have implicated a changing nutritional environment in early postnatal life to beta-cell maturation, with a high amino acid-based nutrition being present before birth to fuel tissue and organ growth in the last third of pregnancy being replaced by a high-fat milk diet during lactation, and the emergence of a temporally regulated carbohydrate-rich intake after weaning (130). The continuation of nutritional restriction or overabundance during pregnancy into the suckling period is likely to alter the maturational timing of pancreatic endocrine cell maturation with potential long-lasting consequences.

The above developmental changes are accompanied by a relative shift in the energy-sensing pathways within the islets away from the amino acid-sensitive mechanistic target of rapamycin (mTOR) mTORC1 complex towards the utilization of AMP-activated protein kinase (AMPK) (131). Failure to undergo this transition resulted in the perpetuation of an immature beta-cell phenotype with poor GSIS. However, the transition in nutrient signaling pathways within the islets is not simply a replacement of mTOR by AMPK, since Raptor, a component of the mTORC1 complex, is central to this process of maturation and Raptor-null beta-cells showed poor insulin release in response to high glucose (132). Deletion of Raptor in Ngn3-expressing beta-cell progenitors caused a decrease in proliferative capacity and the retention of a pool of progenitor cells (133). Consequently, a failure to generate a cohort of mature beta-cells resulted in diabetes by postnatal day 14. Functional maturation of beta-cells is associated with the upregulation of genes characteristic of mature beta-cell function such as glucokinase, *MafA* and urocortin-3 (*Ucn3*) (134–136). The life-long reduction in beta-cell function in the offspring of LP-fed mothers is associated with long-term changes in intracellular signaling pathways such as mTOR. Mouse islets of offspring from mothers that received LP diet during gestation and weaning exhibited decreased levels of activated mTOR protein and impaired glucose and amino acid-stimulated insulin release, which persisted into adulthood (137). The neonatal period is also associated with the expression of transcription factors regulating circadian rhythm, such as the CLOCK gene which controls the expression of oscillatory gene expression in beta-cells (138) and has been reviewed by Chan et al. (139). Such circadian changes in beta-cell function are necessary to accommodate the episodic feeding patterns of adult life. However, how islet circadian rhythms might be altered following exposure to nutritional insults *in utero* is not known.

Beta-cell maturational changes in the neonatal period in rodents are thought to involve changes in beta-cell heterogeneity with evidence of targeted deletions of some populations of beta-cells by apoptosis (140) with other populations, presumably possessing a more maturational phenotype, replacing these. Whether these arise through replication of remaining beta-cells or by neogenesis from resident beta-cell progenitors is unclear, although proliferative progenitors expressing insulin but with poor expression of Glut2 are abundant in the neonatal mouse pancreas (141). Changes in the balance of beta-cells demonstrating mature, functional phenotype during the neonatal period as a result of maternal nutritional insults could have a profound impact on future metabolic control.

High carbohydrate feeding of rat pups shortly after birth alters the developmental trajectory of the endocrine pancreas with pancreatic BCM being increased by postnatal day 12, which pre-disposing the animals to glucose intolerance and obesity as adults compared to receiving normal milk composition pre-weaning (142). Despite an increased insulin secretion pre-weaning on a high carbohydrate diet the mean islet size was reduced with evidence of increased apoptosis of beta-cells, although this was compensated for by increased neogenesis of new beta-cells from the pancreatic ductal epithelium and an overall increase in pancreatic *Pdx1* expression (143, 144). This is suggestive of beta-cell regeneration as a response to the secretory stress of hyperinsulinemia induced by neonatal carbohydrate overnutrition, which is likely to disrupt the normal developmental ontogeny of the endocrine pancreas and predispose to glucose intolerance in adult life. Support for this hypothesis was provided by Li et al. (145) who showed that overnutrition in the neonatal mice inhibited the expression of *MafA*, a key transcription factor for the functional maturation of beta-cells, resulting in glucose intolerance in the adults. The maturational defect may primarily be in the development of acute insulin release mechanisms in response to changing glucose concentrations since a high fat/high carbohydrate feeding regime in neonatal mice with an additional environmental stressor of maternal separation, as also employed by Srinivasan et al. (144) resulted in hyperinsulinemia and glucose intolerance (146).

In the model of maternal undernutrition through LP diet administration to rodents, returning the mother to control diet immediately following parturition partially reversed the reduction in BCM and the longer-term deficiencies in islet function seen in the offspring, but if the LP diet was extended until weaning the changes were irreversible and contributed to adult glucose intolerance (147, 148). The definitive programming of risk of adult metabolic diseases in the neonatal period is thought to involve altered epigenetic control of gene expression. Around 17% of genes in mouse pancreatic islets were found to be differentially expressed between 2 and 10 weeks of age, including an increase in insulin expression, genes involved with calcium gene transport, and AMPK (149). This was associated with histone modifications at transcriptional start sites of over 200 genes. A further developmental window for the programming of future metabolic disorders was found to exist between weaning and puberty as shown by de Oliveira (150) who introduced a LP diet to male rats between postnatal days 30 and 60, before return to a normal rat chow. Food intake and body-weight gain were increased following return to the normal diet and, as adults, the animals demonstrated an increased fasting glucose level, hyperinsulinemia, hyperleptinemia and insulin resistance.

Collectively these findings would suggest that the neonatal changes in beta-cell maturation are an important determinant of longer-term metabolic control, as is the pre-pubertal period. In humans the equivalent beta-cell maturation to that seen in rodent neonates occurs during third trimester (151), although available data is scarce. Both fetal and neonatal developmental processes appear essential for determining the future plastic potential for both islet function and BCM throughout adult life. While changes to pancreatic progenitor cell populations may be altered through nutritional insults in fetal life, their implications may carry forward into neonatal life in terms of appropriate ontological changes in beta-cell maturation in response to environmental triggers such as milk composition. Should nutritional imbalance be continued throughout lactation this will represent an additional stressor that could disrupt the future equilibrium of pancreatic endocrine function. The neonatal period is therefore likely to represent a critical window where nutritional insult can shape the metabolic axis of the offspring, or alternatively a window for therapeutic intervention.

## 5 Mechanisms determining future offspring metabolic health

Maternal overnutrition during pregnancy results in the reprogramming of morphology and function in the pancreas with life-long implications for metabolic health. Administration of a HFD to pregnant rodents resulted in long-term glucose intolerance in the offspring and the appearance of T2D in adulthood (90, 152). This was despite an often-elevated initial insulin secretion in the offspring associated with an increased BCM (153–155). This appears similar to the decline in beta-cell function over time seen in human T2D and involves reduced expression of key beta-cell transcription factors such as *Pdx1* and Neuronal differentiation gene 1 (*NeuroD1*), and genes protective of cellular oxidative stress such as superoxide dismutase 1 (*Sod1*) (101, 153). An increased presence of inflammatory cytokines coupled with elevated free fatty acids associated with childhood obesity have been shown to modify the developmental trajectory of a number of organs including the hypothalamus, adipose tissue, liver and pancreas (156). Maternal obesity during pregnancy has also been linked with extensive epigenetic changes in gene expression. In the mouse, a HFD during pregnancy resulted in extensive hypermethylation of hepatic genes in the offspring including genes with shared function in the endocrine pancreas such as *HNF4α* (157). Similarly, maternal obesity was associated with widespread hypomethylation of genes within the hypothalamus of offspring together with hypermethylation of the insulin receptor promoter, resulting in insulin resistance (158). An analysis of epigenome-wide changes in whole blood from youth with T2D whose mothers had diabetes revealed decreased methylation of PFKFB3 (159). This gene is expressed in beta-cells as part of an injury response in hyperglycemia to switch cells to glycolysis for ATP production and protect them from glucotoxic damage, but at the expense of GSIS. Expression of PFKFB3 was increased in beta-cells from rats and humans with T2D (160). One of the most extensive follow-up studies on the effects of maternal gestational hyperglycemia on future child metabolism has been the HAPO follow-up study. Using a cohort of almost 5,000 children studied at age 10-14 years maternal fasting blood glucose was shown to be positively associated with child glucose tolerance and haemoglobin A1C, and negatively associated with insulin sensitivity (161). Newborn adiposity and cord blood hyperinsulinemia was related to maternal glycemic control and was predictive of childhood adiposity at age 10-14 (96). For those mothers who developed GDM the risk of impaired glucose tolerance in the children was doubled compared to women who did not (162). These associations extend to cardiovascular disorders also since good maternal cardiovascular health at 28 weeks’ gestation was significantly associated with cardiovascular health indices in the children (163).

Maternal undernutrition through feeding of a LP diet in rodents was associated with adult offspring showing increased visceral adiposity and glucose intolerance compared to offspring from control diet-fed dams (115), although this need not lead to greater total body weight gain (164). This was accompanied by impaired GSIS and reduced BCM (90) with the metabolic phenotype being more pronounced in males than in females (165). Deficits in BCM existed at birth but resolved in juvenile animals and reappeared later in adulthood (166). Pancreatic islets of Langerhans isolated from rats born to mothers exposed to LP diet during pregnancy showed increased levels of beta-cell apoptosis when exposed to pro-inflammatory cytokines as compared to controls (167). Also, when young mice or rats were given STZ to reduce BCM a prior *in utero* exposure to maternal LP diet impaired their ability to regenerate beta-cells (168, 169). Both types of study demonstrated the resilience of BCM to metabolic stress to be lowered following fetal undernutrition. We examined the effects of the milder metabolic stress of a subsequent pregnancy on the endocrine pancreas of mice exposed *in utero* to maternal LP diet. Young, sexually mature female offspring exposed to LP diet *in utero* had normal glucose tolerance and BCM prior to mating. However, during pregnancy they developed impaired glucose tolerance and a suboptimal increase in BCM compared to mice born to mothers receiving control protein diet (170). The adaptive deficit in BCM during pregnancy involved a reduction in the rate of beta-cell proliferation and the presence a lower population of Ins^+^ Glut2^-^ beta-cell progenitors (170–172). Glucose tolerance in control-fed mice was no different from pre-pregnancy values following a glucose tolerance test within two weeks following parturition. However, in the offspring of LP-fed dams glucose tolerance did not return to pre-pregnancy values until 3 months post-partum (173). Patients experiencing GDM also experience long-term metabolic challenges long after parturition and have a high risk of subsequently developing T2D (174). The impact of feeding LP diet during gestation on offspring glycemic control has been shown to extend to the F2 generation, at least in mice (175).

Exposure of mice to LP diet prior to weaning resulted in long-lasting decreases in the relative expression of endocrine pancreatic genes associated with cellular respiration, antioxidant expression, gluconeogenesis, regulators of cell proliferation such as insulin-like growth factor-1, and the expression of insulin and proglucagon (176). At a proteomic level, 45 proteins were found to significantly differ in islets isolated from mice experiencing LP diet *in utero* relative to controls, which included proteins involved with mitochondrial function and redox potential, protein synthesis, proliferative cell cycle control, and differentiation (177). Changes in metabolically associated gene expression may result from changes in epigenetic marks involving differential methylation or histone acetylation. This was demonstrated for a progressive epigenetic silencing of *Pdx1* with advancing age in islets from rats who experienced intrauterine growth retardation, resulting in T2D (178). Similarly, maternal undernutrition was associated with altered methylation and histone marks resulting in a reduced expression of *HNF-4α* a transcription factor associated with beta-cell glucose metabolism (179). As with *Pdx1*, the progressive silencing increased with age. Epigenetic changes also occur in insulin sensitive tissues in response to fetal undernutrition, with isolated rat adipocytes demonstrating a higher glucose uptake in the absence of insulin but a diminished insulin-dependent glucose uptake, resulting in a net insulin resistance (180). This involved disrupted insulin signaling through a reduced activation of insulin receptor substrate 2 (IRS2) and the downstream second messenger Akt, with a subsequent reduction in Glut4 cellular cycling activity (181). Epigenetic changes have also been identified in human adipose-derived stem cells from infants with intrauterine growth retardation (IUGR) resulting in a down-regulation of CYCLIN-T2 expression, a gene controlling adipose maturation that confers risk of abnormal lipid metabolism (182).

Underlying mechanisms that predispose to adult obesity following IUGR also involve changes to appetite regulation within the hypothalamus. The arcuate nucleus (ARC) derives metabolic information from hormonal and neural inputs originating in the pancreas, adipose and gastro-intestinal tract. The ARC contains neuropeptide-Y (NPY)-expressing medial orexigenic neurons and pro-opiomelanocortin (POMC)-expressing lateral anorexigenic neurons. Hypothalamic neuronal stem cells develop *in utero* to form the ARC appetite center expressing either orexigenic or anorexigenic peptides. Satiety signals were dysregulated in animals experiencing prior IUGR, resulting in greater food intake postnatally with hyperphagia and altered hypothalamic leptin and ghrelin sensitivity (183). Premature cellular aging has also been postulated to occur in tissues of offspring exposed to maternal undernutrition *in utero* using the surrogate of telomere shortening with age. Hexameric repeat sequences at the end of chromosomes shorten with each subsequent cell division, ultimately resulting is cell senescence, and accelerated shortening has been observed in both rodent models and humans of low birth weight for gestational age (184). In the context of endocrine pancreatic development this may be a contributory mechanism to premature differentiation of functional beta-cells in offspring of mice exposed to LP diet during pregnancy. In the maternal LP diet mouse model, a premature expression of *MafA* was reported within beta-cells due to changes in epigenetic modification of the gene (185). The transcription factor *MafA* is necessary for the gain of acute glucose-stimulated insulin release by beta-cells but is also associated with a decline in proliferative activity. Thus, premature expression of *MafA* due to advanced cellular aging may subsequently limit adult beta-cell mass.

The altered metabolic phenotype in adult offspring who encountered nutritional stress *in utero* has also been linked to changes in maternal glucocorticoid levels and placental transfer to the fetus. Pregnancy is associated with altered glucocorticoid homeostasis due to the production of corticotrophin releasing factor (CRF) by the placenta and release into the maternal circulation, resulting in adrenal hypertrophy (186). This contributes to a greater than five-fold increase in circulating maternal cortisol levels in second and third trimester (187). These levels would be largely transmitted to the fetus if not for the actions of 11beta-hydroxysteroid dehydrogenase -2 (11β-HSD2) in the placenta to degrade cortisol to less biologically active cortisone (188). Treatment of women with an exogenous glucocorticoids that are not degraded by 11β-HSD2, such as dexamethasone, has long-lasting effects on fetal development and pre-dispose to impaired insulin secretion and T2D in the adult offspring (189). Intrauterine growth restriction in human infants is associated with reduced placental expression of 11β-HSD2 resulting in a greater exposure to maternal glucocorticoids (190), which can influence the development of fetal tissues such as the pancreas. Within the fetal rodent pancreas excess glucocorticoids limited pancreatic beta-cell development (191) and had long-term detrimental effects on GSIS in the offspring (192). When simulated through dexamethasone treatment of the pregnant rat the offspring exhibited a relative increase in pancreatic alpha-cell mass and hyperglucagonemia, accompanied by a reduced BCM, islet number and pancreatic insulin content (193). Maternal protein undernutrition restricted to the weaning period in rats similarly resulted in long-term changes in the hypothalamic-pituitary-adrenal axis resulting in elevated corticosterone release in male offspring (194). Hence, an excess of glucocorticoid transfer to the fetus during pregnancy, as can occur in both under – and overnutrition during pregnancy, can reprogram the fetal endocrine pancreas to predispose to adult metabolic dysfunction.

Clearly, maternal overweight and poorer cardiovascular health are major determinants of future child adiposity and metabolic health. The programming effects on the offspring have long-term impacts on glucose tolerance due to insulin resistance. Conversely, maternal gestational undernutrition has long-lasting impact on offspring pancreatic function and insulin-secreting capacity. Epigenetic changes to key genes in the offspring involved in metabolic control associated with either maternal over-nutrition or under-nutrition are long-lasting and may have limited reversibility. This emphasizes the importance of a critical window of optimal maternal nutrition that exists, beginning perhaps earlier than 20 weeks of pregnancy, to avoid a future trajectory of poor metabolic health in the offspring.

## 6 Maternal interventions to reverse future metabolic disease risk in the offspring

The high rates of maternal obesity in the developed world create a risk for a future health burden of chronic diseases in subsequent generations based on pathological development of metabolic pathways in the offspring, including endocrine pancreatic function. Understanding possible temporal windows of therapeutic intervention to prevent or reverse abnormal programming of the metabolic axis in early life is an important topic of study but is limited by the risk of potential adverse drug effects on placental or fetal health. Nevertheless, lifestyle change, nutritional modification and supplements, and pharmaceutics have all been examined as maternal interventions across gestation with endpoints related to pancreatic function, maternal glycemia, the development of GDM, fetal birth weight and adiposity, and subsequent child health. Numerous studies have examined combinations of nutritional change and enhanced physical activity during gestation for obese women to prevent GDM and the risk of LGA offspring (195, 196). The ‘Vitamin D and lifestyle intervention for GDM prevention studies (DALI) recruited pregnant women with a pre-pregnancy BMI of 29 kg/m^2^ or greater before 20 weeks’ gestation and randomly distributed them to healthy eating or increased exercise intervention groups, or both in combination. The combined healthy eating and physical activity group showed significantly lower gestational weight gain compared with a control group but this did not alter maternal fasting glucose, insulin resistance (HOMA-IR) or birth weight, although newborn adiposity and lipid levels were reduced (29, 197, 198). However, there was no change in the number of obese women who subsequently developed GDM. An earlier intervention in pregnancy in the Treatment of Booking Gestational Diabetes Mellitus (TOBOGM) trial did not alter mean birth weight of the offspring but did reduce the number of neonatal complications such as respiratory distress (199). Thus, lifestyle changes alone were largely ineffective at preventing GDM in pregnant women at risk. Metformin is effective in controlling hyperglycemia during pregnancy, however, administration of metformin to pregnant rodents resulted in a reduced offspring birth weight and altered metabolism post-weaning with males developing hyperglycemia, excess adipose and insulin resistance after a HFD challenge, and females showing hypercholesterolemia (200). When used clinically during pregnancy a follow-up study of children born to mothers with GDM found altered fat deposition at two years of age, and greater skinfold thickness at age 9 (201). Possible longer-term effects of metformin use in pregnancy on the function of the endocrine pancreas in the offspring have not been reported.

## 7 Discussion

In summary, nutritional imbalance during pregnancy can contribute to metabolic cellular stress, impaired pancreatic endocrine function, abnormal maternal glycemia, altered placental function and aberrant fetal growth trajectory and birth size. This involves reprogramming of the fetal pancreas and metabolic axis with potentially life-long consequences for the risk of T2D and other metabolic disorders. Altered epigenetic marks affecting both DNA methylation and histone acetylation cause changes in the expression of key transcription factors controlling the set point of glucose sensitivity within pancreatic beta-cells, their ability to survive and recover from metabolic and inflammatory stress, and the appearance of insulin resistance within target tissues with aging. The neonatal period appears to be a key developmental window where there is a potential for reversal of some of these detrimental cellular pathways, particularly within the endocrine pancreas of the offspring. In animal models of under- or over-nutrition the normal developmental remodelling of BCM and function that occurs between birth and weaning in preparation for an adult periodic feeding regime can be irreversibly impaired. In the situation of maternal under-nutrition at least, this is likely to involve long-term changes to the microvasculature of the islets of Langerhans that may become rate-limiting to GSIS with advancing age. A therapeutic targeting of islet vascular density to enhance angiogenesis could be a strategy for rescuing offspring of mothers from increased risk of T2D in adult life. With respect to the future health of mothers who experience obesity-driven gestational diabetes, clinical observations and animal models suggest that the normal reversal of the greater BCM necessary to support pregnancy is delayed, and is linked to increased risk of future T2D. This suggests that a continuation of therapeutic interventions such as insulin or metformin into the post-partum period might be beneficial to future metabolic health.
